# A Case of Severe Left-Ventricular Noncompaction Associated with Splicing Altering Variant in the *FHOD3* Gene

**DOI:** 10.3390/genes13020309

**Published:** 2022-02-07

**Authors:** Roman Myasnikov, Anna Bukaeva, Olga Kulikova, Alexey Meshkov, Anna Kiseleva, Alexandra Ershova, Anna Petukhova, Mikhail Divashuk, Evgenia Zotova, Evgeniia Sotnikova, Maria Kharlap, Anastasia Zharikova, Yuri Vyatkin, Vasily Ramensky, Alexandra Abisheva, Alisa Muraveva, Sergey Koretskiy, Maria Kudryavtseva, Sergey Popov, Marina Utkina, Elena Mershina, Valentin Sinitsyn, Evgeniya Kogan, Olga Blagova, Oxana Drapkina

**Affiliations:** 1 National Medical Research Center for Therapy and Preventive Medicine, 101990 Moscow, Russia; andorom@yandex.ru (R.M.); meshkov@lipidclinic.ru (A.M.); sanyutabe@gmail.com (A.K.); alersh@mail.ru (A.E.); anna.petukhova.96@gmail.com (A.P.); divashuk@gmail.com (M.D.); evgenia.d.zotova@gmail.com (E.Z.); sotnikova.evgeniya@gmail.com (E.S.); kharlapmaria@yahoo.com (M.K.); azharikova89@gmail.com (A.Z.); vyatkin@gmail.com (Y.V.); ramensky@gmail.com (V.R.); abisheva.alex@gmail.com (A.A.); alisa.fbb@gmail.com (A.M.); skoretskiy@gnicpm.ru (S.K.); kudryavtseva6041995@yandex.ru (M.K.); ODrapkina@gnicpm.ru (O.D.); 2 Federal State Budgetary Institution “National Medical Research Center of Endocrinology” of the Ministry of Health of Russia, 115478 Moscow, Russia; swpopov73@gmail.com (S.P.); mv.utkina@ya.ru (M.U.); 3 Moscow Institute of Physics and Technology, 141701 Dolgoprudny, Russia; 4 All-Russia Research Institute of Agricultural Biotechnology, 127550 Moscow, Russia; 5 Faculty of Bioengineering and Bioinformatics, Lomonosov Moscow State University, Lomonosovsky Prospect 27, Building 10, 119991 Moscow, Russia; 6 Medical Research and Educational Center, Lomonosov Moscow State University, 119991 Moscow, Russia; elena_mershina@mail.ru (E.M.); vsini@mail.ru (V.S.); 7 Department of Faculty Therapy, I.M. Sechenov First Moscow Medical University (Sechenov University), 119991 Moscow, Russia; koganevg@gmail.com (E.K.); blagovao@mail.ru (O.B.)

**Keywords:** left ventricular noncompaction, hypertrophic cardiomyopathy, dilated cardiomyopathy, *FHOD3*, exon skipping, intramyocardial fibrosis, heart failure

## Abstract

Left ventricular noncompaction (LVNC) is a highly heterogeneous primary disorder of the myocardium. Its clinical features and genetic spectrum strongly overlap with other types of primary cardiomyopathies, in particular, hypertrophic cardiomyopathy. Study and the accumulation of genotype–phenotype correlations are the way to improve the precision of our diagnostics. We present a familial case of LVNC with arrhythmic and thrombotic complications, myocardial fibrosis and heart failure, cosegregating with the splicing variant in the *FHOD3* gene. This is the first description of *FHOD3*-dependent LVNC to our knowledge. We also revise the assumed mechanism of pathogenesis in the case of *FHOD3* splicing alterations.

## 1. Introduction

Left ventricular noncompaction (LVNC) is a highly heterogeneous primary disorder of myocardial structure. It is known that other primary cardiomyopathies, in particular, hypertrophic cardiomyopathy (HCM), may significantly overlap with LVNC in terms of both clinical features and genetic causes [1,2]. Nevertheless, the prognosis, treatment and follow-up strategies for these conditions are different [3]. The most acceptable and feasible way for clinical specialists to overcome these difficulties is through thorough instrumental diagnostics with high-quality imaging and the accumulation of knowledge about genotype–phenotype correlations.

Among genetic causes of LVNC, the most prominent are variants in the genes encoding sarcomeric proteins [4,5]. Some of those genes, such as *MYH7* and *MYBPC3*, are widely studied, and a considerable body of information exists about phenotypic manifestations of the mutations in them [6]. However, the spectrum of genes involved in sarcomeric assembly, regulation and function is much wider, and for some of them, the genotype–phenotype correlations and functional consequences of different types of mutation are still understudied. One such gene is *FHOD3*, which encodes a cardiospecific regulator of the actin filament assembly. The *FHOD3* gene product participates in cardiac sarcomere formation and thus directly affects the contractile function of the heart [7]. The first assumptions of the causal role of the *FHOD3* gene in inherited cardiomyopathies were made in 2013 [8], and in 2018, convincing evidence of its causality in HCM was provided [9]. However, existing phenotype data from the *FHOD3* variant carriers are scarce, and, to our knowledge, *FHOD3*-positive LVNC patients have not been described yet. 

Here, we present a case of familial LVNC harboring the likely pathogenic variant in the canonical donor splice site in the *FHOD3* gene. We describe the phenotypic features of the affected carriers and diagnostic issues observed in these patients. We also discuss the possible pathogenetic mechanism for splicing alterations in the *FHOD3* gene, given that various alternative splicing events are a well-known mechanism of disease for sarcomeric genes [10,11]; however, for *FHOD3*, the current data are insufficient to conclude confidently about the role of splicing variations.

## 2. Materials and Methods

### 2.1. Clinical Investigation of the Patients

Two adult Russian siblings with noncompaction cardiomyopathy, heart failure, arrhythmia and different complications were admitted to the National Medical Research Center for Therapy and Preventive Medicine. The patients and their available blood relatives (i.e., their mother and the female sibling’s daughter) underwent clinical examination, including collection of venous blood samples and buccal swabs, general and biochemical blood tests, electrocardiography using 24-h Holter monitoring electrocardiogram (HM-ECG), echocardiography and cardiac magnetic resonance imaging (cMRI). The study was performed in concordance with the Declaration of Helsinki in its current form and approved by the Institutional Review Boards of the National Medical Research Center for Therapy and Preventive Medicine (Moscow, Russia). Voluntary informed consent was obtained from all study participants and/or their legal representatives.

### 2.2. Exome Sequencing and Bioinformatic Analysis

**Figure 1:**
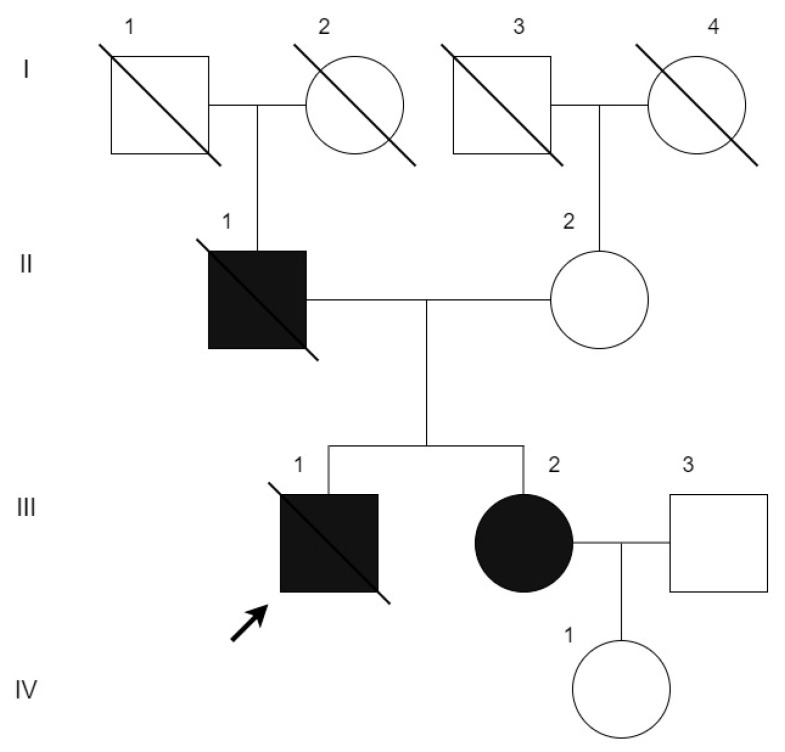
The family tree. Men are shown by squares and women by circles. Black figures indicate affected persons, crossed out figures indicate deceased. The proband is marked by the arrow. For a brief phenotypic summary of family members, see Table 1.

Molecular genetic investigation was carried out at the National Medical Research Center for Therapy and Preventive Medicine as we previously detailed in [12]. The investigated family members (i.e., patients II-2, III-1, III-2 and IV-1, see Figure 1) were subject to whole exome sequencing. We isolated DNA from whole blood and buccal swab samples, then prepared IDT-Illumina TruSeq DNA exome libraries and performed sequencing on NextSeq 550 (Illumina, San Diego, CA, USA) in accordance with the manufacturer’s appropriate protocols. Sequencing reads were aligned to the reference genome (GRCh38) using bwa-mem [13]. Single nucleotide variants were called with GATK 4.2 HaplotypeCaller [14] and annotated using Ensembl Variant Effect Predictor (VEP) [15]. For clinical interpretation, we selected the single nucleotide variants with frequencies in the gnomAD database of <0.5%, or missing in the gnomAD. The interpretation of potentially clinically relevant findings was performed in concordance with current international guidelines [16], considering the specifically modified framework provided by ClinGen for putative loss-of-function variants [17]. 

### 2.3. Sanger Sequencing

To verify the findings by Sanger sequencing, the following oligonucleotides were used: 5′-CGACCATAGACAAGCTGCCC-3′ and 5′-TTTATGCCACAACTGCTCCCT-3′ (the size of resulting PCR product was 256 bp). We performed PCRs in 20 μL of a mixture containing 0.2 mM of each nucleotide, 1× PCR buffer, 20 ng of the DNA, 10 ng of each primer, and 2.5 U of AmpliTaq Gold polymerase (Thermo Fisher Scientific, Waltham, MA, USA). The GeneAmp PCR System 9700 thermocycler (Thermo Fisher Scientific, Waltham, MA, USA) was used, with the following parameters: 95 °C—300 s; 30 cycles: 95 °C—30 s, 62 °C—30 s, 72 °C—30 s; 72 °C—600 s. The obtained amplicons were purified with ExoSAP-IT reagent (Affymetrix, Santa Clara, CA, USA). Sanger reactions were performed using the ABI PRISM BigDye Terminator reagent kit v. 3.1 according to the manufacturer’s protocol. The analysis of the reaction products was performed on an Applied Biosystem 3500 DNA Analyzer (Thermo Fisher Scientific, Waltham, MA, USA).

## 3. Results

The proband (III-1, see Figure 1 and Table 1) was diagnosed with non-obstructive HCM in his childhood and remained under cardiologist’s observation until adulthood. His father was reported as having cardiomyopathy of unknown origin and died suddenly at 47. However, from the age of 18 years, the proband had an active lifestyle without restrictions and no follow-ups or therapy. 

**Figure 2:**
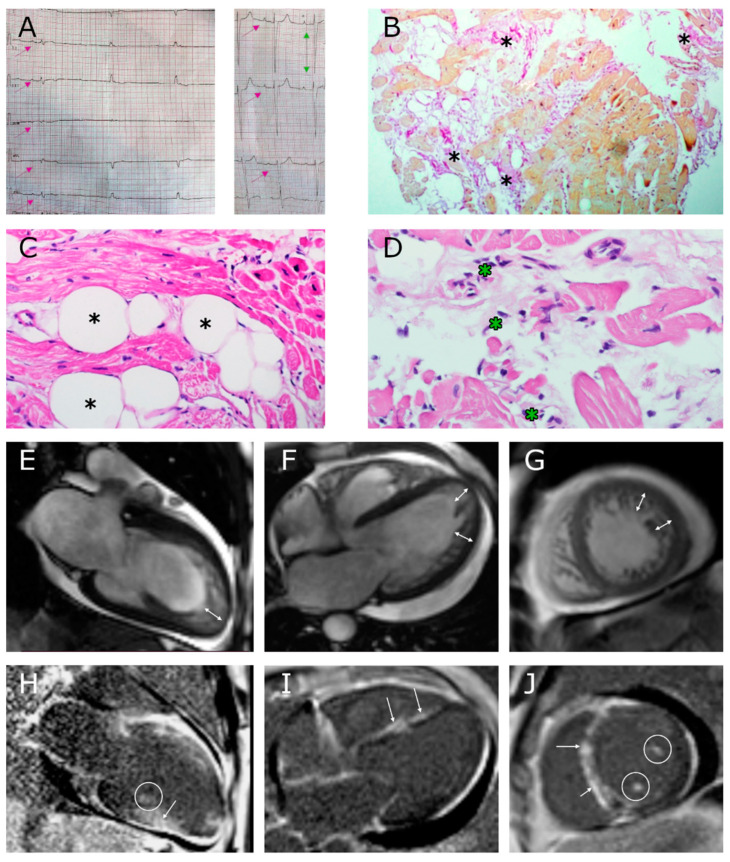
Instrumental data on the proband (III-1). (**A**): The proband’s electrocardiogram, nodal rhythm (pink arrows) and signs of left ventricular hypertrophy (green arrow), recording rate is 25 mm/s. (**B–D**): Endomyocardial right ventricular biopsy specimens, Van Gieson (**B**) and hematoxylin-eosin (**C**,**D**) staining; hypertrophy and dystrophy of cardiomyocytes with cell death, inflammatory lymphocytic infiltration (more than 14 cells per 1 mm square), subendocardial lipomatosis, moderate interstitial fibrosis (marked with asterisks). (**E–G**): Cardiac magnetic resonance imaging (cMRI), SSFP sequence: (**E**) long axis 2-chamber projection, (**F**) long axis 4-chamber projection, (**G**) short axis; double-sided arrows indicate a layer of noncompact myocardium. (**H–J**): cMRI, delayed contrast enhancement, IR sequence with suppression of the signal from the myocardium. Extended areas of intramyocardial fibrosis (non-coronary pattern) in the interventricular septum and lower LV wall are marked with arrows; areas of contrast enhancement in papillary muscles are circled.

He subsequently deteriorated from the age of 44, presenting with an increasing dyspnea, left ventricular dilatation, and heart failure in both circulatory circles. At the time of hospitalization, he had (according to ECHO) left ventricular ejection fraction (LV EF) of 19%, left atrium (LA) size 50 mm, end diastolic diameter (EDD) 51 mm, thrombosis of the LV apex, LV wall thickness 11 mm, and signs of noncompaction myocardium (according to Jenni [18], Stollberger [19] criteria). cMRI with gadolinium showed the signs of noncompaction cardiomyopathy and fibrosis (Figure 2E–J). The electrocardiogram (ECG) showed atrial fibrillation (90–140 beats per minute). The NTproBNP level was 2730 pg/mL, indicating severe heart failure. 

In order to exclude myocarditis as a possible cause of decompensation of heart failure, the patient underwent a myocardial biopsy in the Sechenov University clinic that showed cardiomyocyte hypertrophy and signs of active lymphocytic myocarditis of moderate activity with the death of single cells, severe subendocardial lipomatosis and mild fibrosis (Figure 2B–D). No viral genome was detected in the biopsy sample. Blood tests showed non-significant increase in the level of antibodies to endothelial antigens, cardiomyocytes, and smooth muscles. The patient refused immunosuppressive therapy for myocarditis; he received the standard therapy for heart failure, due to which his condition stabilized. Under dynamic observation in the hospital, his EF reached 24%, and a thrombus in the cavity of the left ventricle was lysed according to the ECHO data. The HM-ECG showed atrial fibrillation and nonsustained ventricular tachycardia. The patient was prescribed amiodarone and beta-blockers to restore the sinus rhythm. However, after leaving the hospital, he soon arbitrarily stopped therapy. At the age of 48, he was hospitalized again due to progression of heart failure. The ECG and HM-ECG showed the threatening heart rhythm deviations, such as sinus bradycardia with episodes of AV block and idioventricular rhythm (Figure 2A). According to ECHO data, EDD was 57 mm, LA 47 mm, and EF 32%. Additionally, LV thrombosis was registered again, and the diagnosis of LVNC was confirmed. It was recommended that the patient undergo implantation of a cardiac resynchronization therapy device (CRT-D), but he refused surgery. The patient had low compliance and ignored the prescriptions after leaving the hospital. At the age of 49, he died due to massive decompensation (it cannot be ruled out whether his death was somehow correlated to severe acute respiratory syndrome coronavirus 2 (SARS-CoV-2) infection). Autopsy was carried out at a distant hospital, but no details are available. 

**Figure 3:**
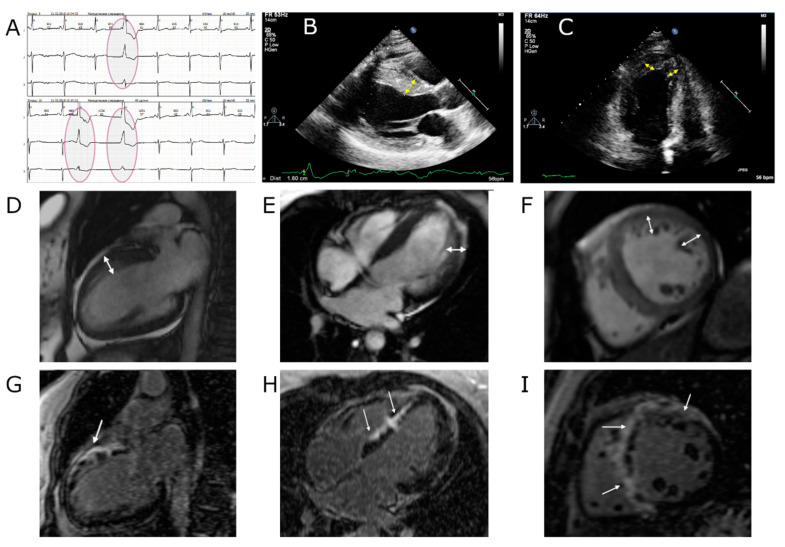
Instrumental data on the proband’s sister (III-2). (**A**): HM-ECG fragment (premature ventricular beats are circled), recording rate is 25 mm/s. (**B**): echocardiographic evidence of moderate interventricular septal hypertrophy (16 mm, shown by a yellow arrow). (**C**): echocardiographic evidence of noncompact LV myocardium (shown by a yellow arrow). (**D–F**): cMRI, SSFP sequence: (**D**) long axis 2-chamber projection, (**E**) long axis 4-chamber projection, (**F**) short axis; double-sided arrows indicate a layer of noncompact myocardium. (**G**–**I**): cMRI, delayed contrast enhancement, IR sequence with suppression of the signal from the myocardium. Extended areas of intramyocardial fibrosis (non-coronary pattern) in the interventricular septum and the anterior LV wall are marked with arrows.

The proband’s sister (III-2, see Figure 1) is currently 44 years old. She had been observed by a cardiologist with a diagnosis of HCM from the age of 10 to adulthood. However, she did not take any medications and had an active lifestyle. At the age of 33, she began to notice the appearance of dyspnea and palpitations during exercise. According to the ECHO, there was an asymmetric hypertrophy of the LV up to 16 mm, increased LV trabeculation, and LV EF 45% (Figure 3B,C).

She was prescribed diuretics and remained after a dynamic examination; her condition remained stable. At the age of 38, cMRI analysis was performed. It revealed the signs of myocardial noncompaction with a noncompacted to compacted layer ratio of 1 to 2.3. The HM-ECG showed a small number of ventricular extrasystoles and a single episode of nonsustained ventricular tachycardia (Figure 3A). She was prescribed bisoprolol 3.75 mg. Despite the ongoing therapy, her condition was worsening. At the age of 40, the cMRI imaging showed marked fibrosis and noncompaction, and EF decreased to 26% (Figure 3D–I). 

The proband’s mother (II-2, see Figure 1) is currently 75 y.o. According to ECHO and cMRI assays, her heart chambers were not expanded, EDD was 51 mm, end diastolic volume (EDV) was 106 mL, EF was 64%, and diastolic dysfunction of type 1 (relaxational) was present. HM-ECG showed normal sinus rhythm with a small number of extrasystoles. In the last few years, she had been suffering from paroxysmal atrial fibrillation that significantly worsened after SARS-CoV-2 infection struck.

**Figure 4:**
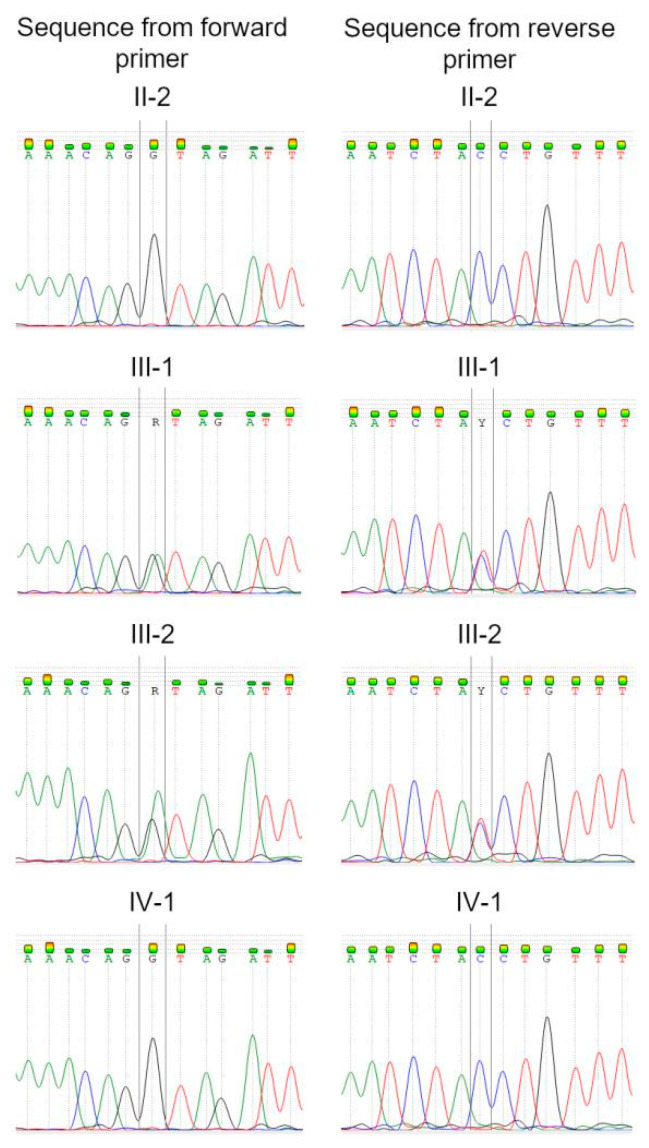
Sanger sequencing electropherograms confirmed *FHOD3* c.1646+1G>A variant for patients III-1 and III-2, and a wildtype sequence for II-2 and IV-1.

To investigate the genetic background of the cardiomyopathy in the reported family, whole exome sequencing on an Illumina platform was performed. Seven rare (MAF < 0.001% according to the gnomAD database) single nucleotide variants were selected for clinical interpretation (Supplementary Table S1). As a result, both in the patient and his sister, we found a variant in the *FHOD3* gene (chr18:36652930 G>A) affecting the canonical donor splice site after exon 12 of cardiospecific isoform (NM_001281740.3: c.1646+1G>A). This variant was absent from the gnomAD population database as well as from the recently emerged open database of genetic variation in Russian population “RuSeq” [20], but it was recently reported by Semsarian et al. [21] in their HCM study. It also had a ClinVar entry describing it as a variant of uncertain significance (VUS) [22] in HCM without further details. The familial screening showed the absence of the variant in unaffected individuals II-2 and IV-1. These findings were validated by Sanger sequencing (Figure 4).

## 4. Discussion

### 4.1. Genotype–Phenotype Correlations

In this study, we observed a remarkable phenotype of noncompaction progressing to heart failure, moderate dilatation and fibrosis in siblings with burdened family history harboring the *FHOD3* splicing variant. To date, no information about genetic findings in the *FHOD3* in patients with LVNC has been published. However, *FHOD3* is a relatively new gene to clinicians, and our knowledge of its genotype–phenotype correlations is still limited. There are only a few studies that describe the clinical features of *FHOD3* variant carriers diagnosed with HCM [9,23,24], and hypertrabeculation is mentioned as an additional finding in these papers. However, we do not have comprehensive information about whether a cMRI was performed for all the reported patients. It should be noted that, in the present case, the proband and his sister were initially diagnosed with HCM, and the diagnosis of LVNC was clarified after the cMRI analysis was performed. This suggests that the true proportion of patients with noncompaction among the *FHOD3* variant carriers may be higher than currently estimated, and that investigation of the *FHOD3* gene in patients with LVNC makes sense. 

One more interesting feature was intramyocardial fibrosis, which is characteristic for patients with pathogenic variants in the sarcomere genes but without left ventricular hypertrophy [25,26,27], but has not been reported before in *FHOD3*-positive patients. Other phenotypic features of our patients, such as arrhythmic complications and decrease in left ventricular ejection fraction, were consistent with the previously described symptoms of *FHOD3*-related cardiomyopathy, thus reinforcing the known genotype–phenotype correlations and further emphasizing the contribution of the revealed genetic variant to the phenotype of affected siblings. 

### 4.2. The Role of Splicing Variants in the FHOD3 Gene

The c.1646+1 substitution in intron 12 of *FHOD3* was described in three unrelated families in an Australian HCM cohort [21], among which one family harbored the c.1646+1 G>C variant and two others had the c.1646+1 G>A substitution. Unfortunately, the authors did not provide a detailed description of their patients’ phenotypes, but their results in combination with our findings provide convincing evidence that the c.1646+1 position is a mutational hot spot in *FHOD3*-related cardiac conditions across populations.

The pathogenetic mechanism this variant acts through is also of interest. Exon 12, which is affected by the c.1646+1 variations, is, first, cardiospecific, and, second, in-frame, i.e., its length (in base pairs) is a multiple of 3. Thus, the most likely [28] consequence of a splicing alteration for this exon—its skipping—is not expected to cause premature translation termination that results in haploinsufficiency of the gene product. 

**Figure 5:**
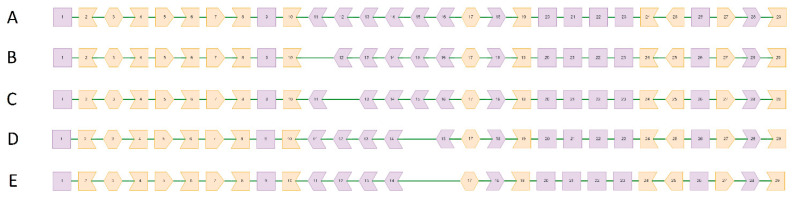
Schematic representation of exonic structure of the wildtype *FHOD3* (transcript NM_001281740.3, cardiac isoform) and its splicing alterations known to date. Each polygon with a number in its center denotes the corresponding exon. The shape of an exon border indicates the reading frame. In-frame exons are colored pink and the rest are colored yellow. (**A**): normal gene product. (**B**): the consequence of c.1286+2 site alteration—lacking exon 11. (**C**): the consequence of c.1646+1 site alterations—lacking exon 12. (**D**,**E**): the consequences of extended deletions described by Ochoa et al. [23]. The picture was drawn using a script written specifically for this article; the design was adopted from Nicolas et al. [31]. Detailed information about the software used for reading frame analysis and illustration can be found in Supplementary Materials.

As *FHOD3* studies in patients with cardiomyopathies have only started recently, there are still few studies that report splicing alterations in this gene. Only six splicing alterations in *FHOD3* have been published so far; two of them are aforementioned changes in position c.1646+1 [21], three are large deletions leading to loss of specific exons [23], and one more is variant c.1286+2delT reported in a cohort study of Chinese patients with HCM [24] without detailed interpretation. We decided to summarize all of these variants and their predicted consequences (see Figure 5) and could see that 100% of known splicing alterations are *in silico* predicted to act through frame-preserving skipping of specific exons from the cardiac isoform of *FHOD3*. This resembles the dominant-negative mechanism through which pathogenic variants in major sarcomeric genes, such as *MYH7*, act [29,30]. Along with the predominance of missense variants in previous *FHOD3* studies, this allows us to speculate that the mechanism of *FHOD3* malfunctioning is dominant-negative rather than haploinsufficiency, although there are still not enough data to make that assertion with confidence. 

### 4.3. Considerations on Management of the FHOD3 Mutants

It is noteworthy that all *FHOD3*-positive HCM patients reported to date have been described as adult-onset cases with rather severe clinical presentation, in some cases with heart failure and/or malignant arrhythmias [23]. However, familial screening undertaken in the same study showed “clinically unaffected” or “probably affected with borderline clinical features” young descendants of the carriers of the pathogenic variants. In the present study, we observed that both affected patients were diagnosed with cardiac structural abnormalities in their childhood, but had no clinical symptoms and thus did not receive any regular follow-up from their young age until they deteriorated. Remarkably, we could not identify any distinct trigger factor of decompensation in our patients. Based on all of the above, we speculate that, at least in some cases, an “adult onset” of the *FHOD3*-related condition may truly be the moment of decompensation that possibly can be avoided if correct follow-up and preventive measures are taken. Thus, we suggest that, for cardiologists, any young or middle-aged patient with findings in *FHOD3* should be the object of close attention, even in cases with mild symptoms.

### 4.4. Limitations of the Study

All of the presented genetic data were based on DNA-level experiments and *in silico* predictions. We were not able to validate our predictions with RNA sequencing or to conduct any functional study of alternative splicing consequences. The cardiac isoform of *FHOD3* (NM_001281740.3) is expressed specifically in the heart, and we would need myocardial biopsy samples to perform a functional study. However, the proband is deceased, and his sister has no clinical indications for a procedure as highly invasive as intramyocardial biopsy. Thus, we could not obtain any relevant samples for the expression study.

## 5. Conclusions

In this work, we observed for the first time the association of an alteration in the *FHOD3* gene with familial LVNC, with prominent myocardium noncompaction as the principal clinical feature. We described the presence of intramyocardial fibrosis cosegregating with the reported variant, thus expanding the spectrum of known genotype–phenotype correlations for the *FHOD3* gene. We also accumulated comprehensive information on all known splicing alterations in this gene and performed an *in silico* analysis of their possible consequences based on sequence properties. We believe that these results will contribute to understanding of the molecular basis of the disease in *FHOD3* mutants. Based on our clinical observations, we assume that *FHOD3*-related cardiomyopathy has a rather poor prognosis and is prone to substantial deterioration, which is probably age-related. Our results underline the difficulty in distinguishing between LVNC and HCM, which may be overcome by, inter alia, the accumulation of clinical observations. 

## Figures and Tables

**Table 1:** The phenotypes of the members of the studied family.

Number in the Family Tree	Phenotype
I-1	unknown
I-2	unknown
I-3	Killed at 25 y.o.
I-4	Died at 89 y.o., cause unknown
II-1	Died at 47 y.o., cardiomyopathy, heart failure
II-2	75 y.o., hypertension
III-1	Died at 49 y.o., noncompaction cardiomyopathy, heart failure, arrhythmia, thromboembolic complication
III-2	44 y.o., noncompaction cardiomyopathy, heart failure, arrhythmia
III-3	43 y.o., unknown
IV-1	11 y.o., healthy

## Data Availability

The data presented in this study are available on request from the corresponding author.

## Supplementary Materials

The following are available online at https://www.mdpi.com/article/10.3390/genes13020309/s1, Table S1: List or rare (MAF < 0.001% according to the gnomAD database) single nucleotide variants identified in the reported family. The script used to illustrate and analyze the position of the reading frame relative to exons (Figure 5) was written specifically for this article and is freely available at https://github.com/SongNightroad/Tips-and-bioinformatics-tools (accessed on 14 December 2021).
