# Supplementary material for: A Case of Severe Left-Ventricular Noncompaction Associated with Splicing Altering Variant in the FHOD3 Gene
Source: Genes (Basel). 2022 Feb 7;13(2):309. doi: 10.3390/genes13020309 (PMC8872028; doi:10.3390/genes13020309)
Supplement: Supplementary file 1 [file genes-13-00309-s001.zip › genes-1534513-supplementary.pdf]

Supplementary Table S1. List or rare (MAF < 0,001% according to the gnomAD database) single nucleotide variants identified in the reported family.

Position (hg38)	Gene	Transcript	cDNA change	Amino acid change	MAF	II-2	III-1	III-2	IV-1	Pathogenicity class	ACMG criteria*
chr2:73448907	*ALMS1*	NM_015120.4	c.2380G>T	p.Ala794Ser	NA	0/0	0/0	0/1	0/1	LB	PM2, BP4, BS4
chr5:128336003	*FBN2*	NM_001999.4	c.3709C>T	p.Arg1237Cys	0,0000197	0/0	0/0	0/1	0/1	VUS	PM2, PP3, BS4
chr8:143932128	*PLEC*	NM_201384.3	c.2082+2T>G		NA	0/0	0/0	0/1	0/0	VUS	PM2, BS4
chr12:21925942	*ABCC9*	NM_020297.4	c.406G>A	p.Ala136Thr	NA	0/0	0/1	0/1	0/0	VUS	PM2, BP4
chr18:36652930	*FHOD3*	NM_001281740.3	c.1646+1G>A		NA	0/0	0/1	0/1	0/0	LP	PM2, PM4, PM1, PP5

* Abbreviations for criteria are provided in accordance with Richards et al. [16] and mean the following: PM1, located in a mutational hot spot and/or specific functional domain; PM2, absent or met at extremely low frequency in large poputational databases; PM4, protein length changes due to in-frame deletions/insertions; PP3, multiple lines of *in silico* evidence indicate deleteriousness; PP5, reported as pathogenic by an independent reputable source; BS4, lack of cosegregation with the disease in the family; BP4, multiple lines of *in silico* evidence show absence of deleterious effects.

LB, likely benign; LP, likely pathogenic; VUS, variant of uncertain significance. 0/0 indicates the wildtype genotype, 0/1 indicates the presence of the variant in heterozygous state.
